# Bioaugmentation of Soil Contaminated with Azoxystrobin

**DOI:** 10.1007/s11270-016-3200-9

**Published:** 2016-12-09

**Authors:** Małgorzata Baćmaga, Jadwiga Wyszkowska, Jan Kucharski

**Affiliations:** Department of Microbiology, University of Warmia and Mazury, Olsztyn|Plac Łódzki 3, 10-727 Olsztyn, Poland

**Keywords:** Azoxystrobin, Soil, Bioaugmentation, Microorganisms, Degradation, Enzymes

## Abstract

The presence of fungicides in the natural environment, either resulting from deliberate actions or not, has become a serious threat to many ecosystems, including soil. This can be prevented by taking appropriate measures to clear the environment of organic contamination, including fungicides. Therefore, a study was conducted aimed at determining the effect of bioaugmentation of soil exposed to azoxystrobin on its degradation and activity of selected enzymes (dehydrogenases, catalase, urease, acidic phosphatase, alkaline phosphatase). A model experiment was conducted for 90 days on two types of soil: loamy sand (pH_KCl_—5.6) and sandy loam (pH_KCl_—7.0), which were contaminated by azoxystrobin at 22.50 mg kg^−1^ DM of soil and inoculated with a specific consortium of microorganisms. Four strains of bacteria were used in the experiment (*Bacillus* sp. LM655314.1, *B. cereus* KC848897.1, *B. weihenstephanensis* KF831381.1, *B. megaterium* KJ843149.1) and two strains of mould fungi (*Aphanoascus terreus* AB861677.1, *A. fulvescens* JN943451.1). Inoculation of soil with the consortium of microorganisms accelerated the degradation of azoxystrobin. The isolated microorganisms were more active in loamy sand because within 90 days azoxystrobin was degraded by 24% (*Bacillus* sp., *B. cereus*, *B. weihenstephanensis*, *B. megaterium*) to 78% (*Aphanoascus terreus*, *A. fulvescens*). In sandy loam, azoxystrobin was degraded by 9% (*Aphanoascus terreus*, *A. fulvescens*) to 29% (*Bacillus* sp., *B. cereus*, *B. weihenstephanensis*, *B. megaterium* and *Aphanoascus terreus*, *A. fulvescens*). The activity of soil enzymes was also changed as a result of inoculation of soil with microorganisms. The activity of all of the enzymes under study was found to have increased when soil augmentation was performed.

## Introduction

Fungicides are organic contaminants which have received considerable attention due to their presence in different ecosystems. Depending on the type of compound and the environmental factors, their decomposition occurs at varying rates and the intermediate products can pose a greater threat to living organisms (Cooper and Dobson 2007). Joly et al. (2015) reported that some soil properties, i.e. pH, moisture content, structure, and content of organic matter, can directly affect the bioavailability of the substances and, in effect, their degradation. In a biological sense, a chemical compound is bioavailable when it can be taken up by an organism or when it can cause a toxic effect. On the other hand, from a chemical perspective, a substance is bioavailable if, in a specific environmental conditions and during a specific time, it is desorbed from soil to soil solution (Semple et al. 2003). Sorption of fungicides by silty and mineral soil fractions is one the of most important processes which affect the bioavailability of fungicides (Joly et al. 2015). Some fungicides can be bound strongly by soil, which prevents their desorption and decreases their bioavailability (Adettutu et al. 2008). Due to the seasonality of fungicidal diseases, fungicides can be used several times during one growing period in one area. Continual and regular application of fungicides can contribute to their accumulation in soil, which results in negative changes in the functions of the ecosystem (Damalas et al. 2006; Jastrzębska and Kucharski 2007). This can manifest itself by a decrease in the number and biodiversity of microorganisms and, in consequence, their biological activity (Baćmaga et al. 2015). However, some microorganisms can become considerably resistant to such agents by developing appropriate mechanisms. They develop such resistance by exchange of their genetic material and by mutations and selection. Microorganisms with such features are effective in the degradation of pesticides in soil, owing to which they can be used in the bioremediation of areas heavily contaminated with fungicides (Komárek et al. 2010). Microorganisms which are adapted to adverse environmental conditions are able to neutralise such chemical substances by using them as a source of construction and energy substrates (Ahemad and Khan 2012; Cea et al. 2010). Transformation of pesticides catalysed by enzymes produced by living organisms takes place and results in changes in their structure and toxicological properties (Vryzas et al. 2012). Bioaugmentation is one of the most common techniques used in clearing the soil environment of different types of contaminants. It involves introducing to the soil specialised autochtonic or allochtonic microorganisms to remove organic compounds resistant to degradation (Zhao et al. 2009). To make bioaugmentation effective, such microorganisms must be able to transform specific compounds (Ma et al. 2015). Moreover, bioaugmentation of soil with a consortium of microorganisms is more effective in the decomposition of fungicides than using single strains (Zhang et al. 2012). Microbiological decomposition of fungicides is regarded as one of the main processes which determines what happens to them in the environment. Using microorganisms in detoxication and degradation of organic contaminants is increasingly often used in bioremediation of soil contaminated by pesticides (Singh et al. 2006). According to Clinton et al. (2011), the most significant role in the transformation of fungicides, including representatives of strobilurin group, is assigned to soil microorganisms. Therefore, some fungicide degradation products may constitute a good energetic substrate for soil microorganisms, which leads to their increased numbers and activity and further improves soil biological properties.

Therefore, the aim of this study was to evaluate the degradation potential of microorganisms isolated from soil contaminated with azoxystrobin in the process of bioremediation of soil exposed to this fungicide. The effect of bioaugmentation on the soil microbiome was also analysed, based on the basis of the activity and resistance of soil enzymes.

## Material and Experimental Methods

### Experimental Design and Procedure

The experiment was conducted under laboratory conditions. Since the type of soil is of considerable importance in the pesticide degradation process (including fungicides and bioremediation), two soils with different granulometric composition and physicochemical properties were used (loamy sand and sandy loam). One hundred grams of air-dry soil was weighed and transferred to 150 cm^3^ beakers. Aqueous emulsion of azoxystrobin at 22.50 mg kg^−1^ DM of soil was added in three replications to each beaker with soil. Azoxystrobin was added to the soil as Amistar 250 SC formula. Subsequently, the soil was inoculated with an appropriate consortium of microorganisms by adding 1 cm^3^ of microorganisms suspension, which gave 10^9^ of cells kg^−1^ DM of soil. In the case of bacteria: *Bacillus* sp. LM655314.1 constituted 22%; *B. cereus* KC848897.1 constituted 29%, *B. weihenstephanensis* KF831381.1 constituted 25%, *B. megaterium* KJ843149.1–24%, whereas in the case of fungi: *Aphanoascus terreus*, AB861677.1 constituted 55% and *A. fulvescens* JN943451.1 constituted 45%. In the case of application of bacterial and fungal consortium, bacteria constituted 56%, while fungi 44%. Soil without the fungicide and a consortium of microorganisms was used as a control. The soil was mixed thoroughly, brought to 50% of capillary water capacity with sterile demineralised water and subsequently incubated at a constant temperature (25 °C) for 90 days. The moisture content in the soil samples was kept at the same level throughout the incubation period and evaporating water was made up regularly. After 90 days of the experiment, residues of azoxystrobin and the activity of soil enzymes were determined in soil samples. The following combinations were prepared in the experiment: (1) control (soil with no fungicide or consortium of microorganisms), (2) soil with azoxystrobin, (3) soil with azoxystrobin and consortium of bacteria, (4) soil with azoxystrobin and consortium of fungi, and (5) soil with azoxystrobin and consortium of bacteria and fungi.

### Characteristics of the Soil Material

Laboratory tests were carried out on two soils classified as Eutric Cambisols (WRBSR World Reference Base of Soil Resources 2014), which—according to their granulation—can be classified as loamy sand and sandy loam. The soils are characterised in Table 1. The soils were collected from the arable humic level (depth 0–20 cm) on land owned by the Teaching and Experimental Station in Tomaszkowo near Olsztyn (north-east of Poland, 53.71610 N, 20.41670 E). Before the experiment was set up, the following were determined: granulometric composition of the soils (particle size was determined with a Mastersizer 2000 laser metre), pH, hydrolytic acidity, total exchangeable bases, organic carbon and total nitrogen by the procedure described in the paper by Carter and Gregorich (1993) and the content of exchangeable cations (K^+^, Na^+^, Ca^2+^, Mg^2+^) by the method described by Harris (2006).

**Table 1:** Characteristics of soils used in the experiment

Parametr	Loamy sand (ls)	Sandy loam (sl)
sand (2000–50 μm) %	80.50	69.41
silt (50–2 μm) %	18.00	17.03
clay (<2 μm) %	1.50	13.56
pH_KCl_	5.60	7.00
HAC (mmol^(+)^ kg^−1^)	18.66	6.40
TEB (mmol^(+)^ kg^−1^)	40.00	165.9
C_org_ (g kg^−1^)	10.00	14.30
N_total_ (g kg^−1^)	0.58	0.98
Content of exchangeable cations (mg kg^−1^)		
K^+^	217.73	180.00
Ca^2+^	568.60	2571.40
Na^+^	100.34	20.00
Mg^2+^	54.52	59.50

pH_KCl_—soil reaction, HAC—hydrolytic acidity, TEB—sum of exchangeable bases, C_org_—organic carbon content, N_total_—total nitrogen content, content of exchangeable cations: K^+^, Na^+^, Ca^2+^, Mg^2+^

### Characteristics of the Fungicide

Amistar 250 SC was manufactured by Syngenta. It is used to control fungal diseases of crops and ornamental plants. The dose recommended by the manufacturer ranges between 0.8 and 1.0 dm^3^ ha^−1^. Its active substance is azoxystrobin (250 g dm^−3^), which is one of strobilurins. Azoxystrobin (methyl (*E*)-2-{2-[6-(2-cyanophenoxy)pyrimidin-4-yloxy]phenyl}-3-methoxyacrylate) is a highly active antifungal agent which inhibits mitochondrial respiration (Adettutu et al. 2008). The half-life of azoxystrobin in soil can range from 14 days to 6 months (Rodrigues et al. 2013).

### Preparation of Microorganism Inoculums

This study employed strains of microorganisms which were isolated from typical brown soil contaminated with azoxystrobin at 22.50 mg kg^−1^ DM of soil. In order to obtain pure typical colonies of microorganisms, they were cultured and then purified by multiple passages onto microbiological media. The bacteria were identified on the basis of the nucleotide sequence from the analysis of gene 16S rRNA, and fungi—based on an analysis of the ITS region. A more detailed procedure of isolation and identification of microorganisms was described in the paper by Baćmaga et al. (2015). The microorganism strains (*Bacillus* sp. LM655314.1, *B. cereus* KC848897.1, *B. weihenstephanensis* KF831381.1, *B. megaterium* KJ843149.1, *Aphanoascus terreus* AB861677.1, *A. fulvescens* JN943451.1), whose growth is the most intensive in soil contaminated with azoxystrobin, were used in soil bioaugmentation. Suspension of microorganisms was obtained by transferring the isolates with 0.85% NaCl onto sterile microbiological media. The microorganisms were subsequently multiplied in an INE 200–800 incubator manufactured by Memmert Perfect at a constant temperature of 28 °C for 72 h. The bacteria were cultured on the PCA medium and the mould fungi were cultured on the Sabourauda medium. The composition of the media used in the study was presented in the paper by Baćmaga et al. (2015).

### Analysis of Azoxystrobin Residue in Soil

Azoxystrobin residue in soil was determined by the method described in the paper by Łozowicka et al. (2016). The chemical analyses were carried out with an Agilent 7890A gas chromatographic unit (Santa Clara, California, USA). Data processing was effected with Chemstation software (Hewlett-Packard, version A.10.2). Fungicide residue was determined by comparing the retention between soil samples and applicable standards.

### Determination of Activity and Resistance of Soil Enzymes

Enzymatic analyses (in three replications) were carried out after 90 days of incubation. The activity of five soil enzymes was determined: dehydrogenases (Öhlinger 1996), catalase and urease (Alef and Nannipieri 1998) as well as acidic phosphatase and alkaline phosphatase (Alef et al. 1998). A detailed methodology of determining the soil enzyme activity was given in the paper by Kucharski et al. (2016). The activity of the enzymes mentioned above was used to determine their resistance index (RS) for contamination with azoxystrobin of soils in which bioaugmentation was performed by the method described by Orwin and Wardle (2004).

### Statistical Analyses of Results

The results were worked out by an analysis of variance ANOVA (*p* = 0.01) with Statistica 12.5 software (StatSoft, Inc. Statsoft and Statistica 2015). Homogeneous groups were identified with a two-way analysis of variance ANOVA using Tukey’s range test at the significance level of *p* = 0.01. The resistance of enzymes in soil contaminated with azoxystrobin was analysed by means of the principal component analysis (PCA) with multi-dimensional exploration techniques. The percentage share of observable variability was calculated using the η^2^ index with the ANOVA variance analysis method at *p* = 0.01.

## Results and Discussion

### Biodegradation of Azoxystrobin in Soil

**Fig. 1:**
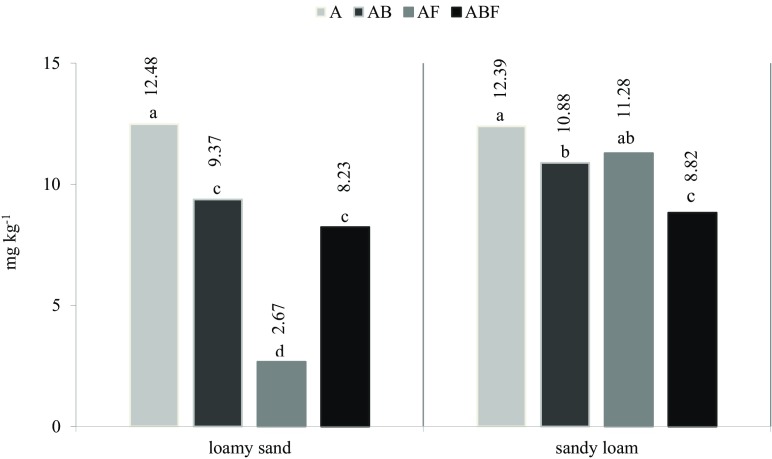
Azoxystrobin residue in soil after bioaugmentation, mg kg^−1^ DM of soil. **A** Azoxystrobin, **B** Consortium of bacteria (*Bacillus* sp. LM655314.1, *B. cereus* KC848897.1, *B. weihenstephanensis* KF831381.1*, B. megaterium* KJ843149.1), **F** Consortium of fungi (*Aphanoascus terreus* AB861677.1, *A. fulvescens* JN943451.1). Homogeneous groups were identified by the same letters (two-way analysis of variance ANOVA by means of Tukey’s range test at the significance level of *p* = 0.01; first factor—type of soil, second factor—type of microorganism consortium)

Microbiological degradation is one of the main processes by which fungicides diminish in soil. Even the most stable compounds are transformed by microorganisms to less-toxic forms (Bending et al. 2007). According to Castillo et al. (2006), degradation of pesticides is a multi-stage process, which is effected by a consortia of microorganisms, i.e. by enzymes produced by such microorganisms. The microorganisms isolated from soil contaminated with azoxystrobin in this study had a high degradation potential compared to the fungicide. Their degradation capacity largely depended on the type of soil as well as on the consortium of microorganisms applied. The statistical analysis of the effect of the observable variability factors η^2^ (Table 2) found that the type of the applied microorganism consortium had the greatest effect on the rate of azoxystorbin degradation in the soil (45%), while the type of used soil modified this rate to a lesser degree (20%). Degradation of azoxystrobin was faster in soil inoculated with microorganisms than in non-inoculated soil (Fig. 1). During the 90 days of the experiment, azoxystrobin 45% was degraded in soil without the inoculum both in loamy sand and in sandy loam. This may have been caused by inhibition by azoxystrobin of the activity of microorganisms which naturally occur in soil. The highest potential for degradation of azoxystrobin in loamy sand was shown by fungi, which degraded it by 88%. On the other hand, transformation of azoxystrobin in sandy loam was the fastest when a consortium of bacteria and fungi was added (degradation of azoxystrobin at 61%). Considering the type of soil, it was shown that the effect of bioaugmentation was greater in loamy sand. Adding a mixture of microorganisms to this soil resulted in faster degradation of azoxystrobin. Soils containing less organic matter and silty fractions can adsorb fewer pesticides on soil colloids, which makes the preparations more available to microorganisms (Adettutu et al. 2008). Singh et al. (2006) reported that inoculation of soil with *Enterobacter asburiae* B-14 accelerated degradation of fenamiphos and chlorpyriphos. Ahmad et al. (2012) carried out a study with chlorpyriphos and introduced the strain of *Bacillus pumilus* C2A1 to soil. Inoculation of soil with these bacteria had a beneficial effect on degradation of the tested insecticide and production of plant biomass. The microorganisms degraded chlorpyriphos by 97% over 45 days. These results were similar to the findings of Abd-Alrahman and Salem-Bekhit (2013) who used a strain of *Psedomonas alcaligens* bacteria to degrade butachlor. They showed that this bacteria degraded butachlor (50 mg kg^−1^ DM of soil) by 75% over 21 days. Sankaran et al. (2010) reported that mould fungi are very effective in eliminating pesticides. This results from their high species diversity, resistance to contamination and ability to produce enzymes which take part in degradation of the compounds. According to the report by Ma et al. (2015), fungi can grow in extreme environmental conditions (e.g. changes of temperature, humidity and pH of soil as well as nutrient depletion), owing to which they can play a huge role in eliminating organic contamination, including fungicides. Bhalerao (2012) showed the strain of *Aspergillus niger* ARIFCC 1053 to be highly effective in the degradation of endosulphane. Furthermore, a study carried out by Abd-Alrahman and Salem-Bekhit (2013) revealed that *Trichoderma viride* degraded butachlor by 98% over 15 days. An earlier study by Kodama et al. (2001) showed that the strain of *Penicilium steckii* DS6F degraded simazine applied at 25 and 50 mg dm^−3^ by 53% during 5 days. In this study, bioaugmentation of soil with selected strains of microorganisms also contributed to an increase in the degradation of azoxystrobin, its degradation also depended largely on the type of soil and the inoculum applied.

**Table 2:** Percentage share of factors for observable variability η^2^

Factors	Deh	Cat	Ure	Pac	Pal	DA
TS	88.19	78.12	75.38	89.21	85.41	20.31
TM	9.658	15.34	19.23	6.291	11.79	45.41
TS · TM	2.026	6.385	3.880	3.591	2.475	34.19
Error	0.126	0.155	1.510	0.908	0.325	0.090

TS—type of soil, TM—type of microorganism consortium, Deh—dehydrogenases, Cat—catalase, Ure—urease, Pac—acid phosphatase, Pal—alkaline phosphatase, DA—degradation of azoxystrobin

### Activity of Enzymes and their Resistance to Azoxystrobin

**Fig. 2:**
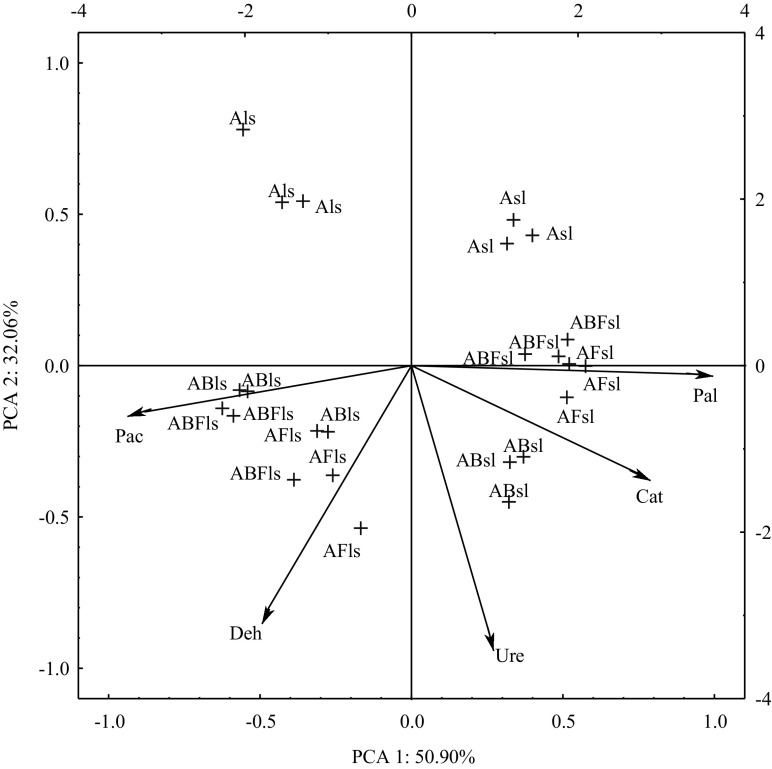
Effect of bioaugmentation on soil contaminated with azoxystrobin on the index resistance of enzymes. ls—loamy sand, sl—sandy loam, **A** azoxystrobin, **B** consortium of bacteria (*Bacillus sp*. LM655314.1, *B. cereus* KC848897.1, *B. weihenstephanensis* KF831381.1, *B. megaterium* KJ843149.1), **F** consortium of fungi (*Aphanoascus terreus* AB861677.1, *A. fulvescens* JN943451.1); Deh—dehydrogenases, Cat—catalase, Ure—urease, Pac—acid phosphatase, Pal—alkaline phosphatase

In order to reduce the adverse effect of pesticides on the environment, microorganisms are used increasingly often in degradation and detoxication of such contaminants. It is noteworthy that microorganisms can adapt to extreme conditions and they can produce enzymes which participate in the transformation of pesticides (Cea et al. 2010; Komárek et al. 2010). Soil is a reservoir of a large amount of enzymes and therefore it is regarded as an indicator of microbiological activity (Baćmaga et al. 2015; Kucharski et al. 2016). Enzymes which indicate soil quality include oxydoreductases and hydrolases due to their participation in a range of biochemical reactions which take place in the environment (Mayanglambam et al. 2005). Soil enzyme activity was modified by both the soil type and the applied microorganism consortium. However, the type of soil had a greater effect on enzyme activity since the percentage share of the observable variability was from 78 to 89% (Table 2). This is why the effect of bioaugmentation on soil contaminated with azoxystrobin on the activity of selected enzymes was assessed. In this study, inoculation of soil with isolated strains of microorganisms caused changes in the activity of soil enzymes (Table 3). Both in loamy sand and in sandy loam, the activity of catalase increased in soil contaminated with azoxystrobin inoculated with microorganisms compared to the soil with the contaminant but without the microorganisms. The greatest effect was observed in loamy sand. The activity of dehydrogenases in loamy sand in which the consortium of bacteria was applied increased by 44% compared to the soil with no azoxystrobin contamination and it increased by 15% in sandy loam. It was noted that the activity of urease increased by as much as 75% compared to the soil contaminated with azoxystrobin in loamy sand after the consortium of bacteria was introduced. The activity of urease in sandy loam was the highest after it was inoculated with a consortium of bacteria and fungi (an increase in urease activity by 49%). The effect of microorganisms in the case of phosphatases varied. The activity of acidic phosphatase increased by 5.9% in loamy sand in which a consortium of bacteria and fungi was applied compared to the soil contaminated with azoxystrobin, and it increased by 8% in sandy loam after inoculation with bacteria. Inoculation of loamy sand with a mixture of fungi (*Aphanoascus terreus* and *A. fulvescens*) resulted in a twofold increase in the activity of alkaline phosphatase compared to soil with no addition of the microorganisms. In turn, the activity of alkaline phosphatase in sandy loam increased by 9% after introducing bacteria (*Bacillus sp*., *B. cereus*, *B. weihenstephanensis*, *B. megaterium*). It can be concluded from the increase in the activity of soil enzymes that the consortia of microorganisms introduced to soil with an addition of azoxystrobin adapted easily to the environment. An increase in the activity of soil enzymes may have also resulted from an increased rate of the fungicide mineralisation after the soil was inoculated with the consortium of microorganisms. Products of pesticide degradation are frequently absorbed by soil microorganisms and used as nutrients (Hussain et al. 2009), which may have stimulated microorganism multiplication in this study and, consequently, increased the soil enzymatic activity. An increase in the activity of soil enzymes can be attributed to changes in the microorganism community, which contributed to neutralisation of the negative impact of azoxystrobin on the biochemical properties of soil. Determination of the enzymatic activity helped to assess the effect of bioaugmentation of soil contaminated with azoxystrobin. Bhalerao (2012) assessed the effect of inoculation with *Aspergillus niger* ARIFCC 1053 of soil exposed to endosulphan by monitoring changes in the activity of dehydrogenases and arylsulphatase. As in this study, inoculation of soil with microorganisms contributed to an increase in the activity of the enzymes under study. This study also determined the resistance of enzymes to contamination with azoxystrobin of soil inoculated with microorganisms (Fig. 2). The values suggest that the first principal component explains 51% of the total variance and the other explains 32%. The distances between vectors which represent primary variables for enzymes and the cases indicate the varied impact of inoculation of soils exposed to azoxystrobin. Increased resistance of dehydrogenases, catalase, urease and alkaline phosphatase to the formula under study was observed after the consortium of fungi was introduced to loamy sand. On the other hand, inoculation of sandy loam with the consortium of bacteria increased the resistance of all of the soil enzymes under study. The results have shown that bioaugmentation is an effective method used in remediation of land contaminated with fungicides.

**Table 3:** Effect of bioaugmentation of soil contaminated with azoxystrobin on the activity of enzymes, kg^−1^ DM of soil h^−1^

Type of soil	Type of microorganisms consorcium	Dehydrogenases μMol TPF	Catalase Mol O_2_	Urease mMol N-NH_4_	Acid phosphatase	Alkaline phosphatase
					mMol PNP	
ls	C	3.273^de^	0.079^cde^	0.485^e^	3.376^ab^	0.721^c^
	A	2.456^f^	0.066^e^	0.307^f^	3.361^ab^	0.227^e^
	AB	3.544^d^	0.090^cd^	0.537^d^	3.315^b^	0.236^e^
	AF	3.196^de^	0.073^de^	0.443^f^	3.331^ab^	0.452^d^
	ABF	3.108^e^	0.095^c^	0.478^e^	3.558^a^	0.132^f^
sl	C	9.864^a^	0.305^a^	1.262^b^	2.593^c^	6.017^ab^
	A	7.755^c^	0.279^b^	0.898^c^	2.183^d^	5.655^ab^
	AB	8.896^b^	0.298^a^	1.280^b^	2.369^c^	6.170^a^
	AF	7.851^c^	0.295^ab^	1.318^a^	2.156^d^	5.458^b^
	ABF	7.875^c^	0.280^b^	1.338^a^	2.149^d^	5.953^ab^

ls—loamy sand, sl—sandy loam, C—soil without an addition of azoxystrobin and a microorganism consortium, A—azoxystrobin, B—consortium of bacteria (*Bacillus sp*. LM655314.1, *B. cereus* KC848897.1, *B. weihenstephanensis* KF831381.1, *B. megaterium* KJ843149.1), F—consortium of fungi (*Aphanoascus terreus* AB861677.1, *A. fulvescens* JN943451.1). The same letters for an enzyme in columns are assigned to the same homogeneous groups (two-way analysis of variance ANOVA by means of Tukey’s range test at the significance level of *p* = 0.01; first factor—type of soil, second factor—type of microorganism consortium)

## Conclusions

Interest in the impact of fungicides on the natural environment is increasing because they are commonly used. The ability of the compounds to accumulate in the environment and in living organisms necessitates seeking different solutions and measures aimed at their effective elimination from the environment. Fungi (*Aphanoascus terreus*, *A. fulvescens*) were the most effective in the degradation of azoxystrobin in loamy sand, while a combination of bacteria and fungi (*Bacillus* sp., *B. cereus*, *B. weihenstephanensis, B. megaterium* and *Aphanoascus terreus*, *A. fulvescens*) degraded azoxystrobin most effectively in sandy loam. Considering the type of soil studied, it was observed that bioaugmentation was more effective in loamy sand than in sandy loam. The introduction of a microorganism consortium into soils also stimulated the activity of the studied enzymes. Bioaugmentation increased the enzymatic activity of soil and accelerated the degradation of azoxystrobin compared to soils with no consortium of microorganisms added. In general, bioaugmentation of soil creates favourable conditions for effective elimination of azoxystrobin and alleviation of the negative effect of the substance on soil microbiome. It also allows one to use microorganisms isolated from soil contaminated with fungicides in the bioremediation of soils exposed to strobilurin fungicides, e.g. azoxystrobin. Finally, bioaugmentation of soils contaminated with azoxystrobin is an effective measure which improves soil microbiological properties.
